# ROBINA: Rotational Orbit-Based Inter-Node Adjustment for Acoustic Routing Path in the Internet of Underwater Things (IoUTs)

**DOI:** 10.3390/s21175968

**Published:** 2021-09-06

**Authors:** Umar Draz, Sana Yasin, Tariq Ali, Amjad Ali, Zaid Bin Faheem, Ning Zhang, Muhammad Hasan Jamal, Dong-Young Suh

**Affiliations:** 1 Department of Computer Science, Lahore Campus, CUI, Lahore 54000, Punjab, Pakistan; sheikhumar520@gmail.com (U.D.); Amjad.ali@cuilahore.edu.pk (A.A.); mhjamal@cuilahore.edu.pk (M.H.J.); 2 Department of Computer Science, University of Sahiwal, Sahiwal 57000, Punjab, Pakistan; 3 Department of Computer Science, University of Okara, Okara 56300, Punjab, Pakistan; sana.yasin@uo.edu.pk; 4 Department of Computer Science, Sahiwal Campus, CUI, Sahiwal 57000, Punjab, Pakistan; tariqali@cuisahiwal.edu.pk; 5 Department of Computre Engineering, University of Engineering and Technology, UET, Texila 47080, Punjab, Pakistan; zaid_fahim@yahoo.com; 6 Electrical and Computer Engineering, University of Windsor, Windsor, ON N9B 3P4, Canada; Ning.Zhang@uwindsor.ca; 7 College of Electronics and Convergence Engineering, Kyung Hee University, Yongin 446-901, Korea

**Keywords:** IoUTs, ROBINA, PA-ROBINA, PL-ROBINA, void node, energy consumption, end-to-end delay, path loss, routing path

## Abstract

The Internet of Underwater Things (IoUTs) enables various underwater objects be connected to accommodate a wide range of applications, such as oil and mineral exportations, disaster detection, and tracing tracking systems. As about 71% of our earth is covered by water and one-fourth of the population lives around this, the IoUT expects to play a vital role. It is imperative to pursue reliable communication in this vast domain, as human beings’ future depends on water activities and resources. Therefore, there is a urgent need for underwater communication to be reliable, end-to-end secure, and collision/void node-free, especially when the routing path is established between sender and sonobuoys. The foremost issue discussed in this area is its routing path, which has high security and bandwidth without simultaneous multiple reflections. Short communication range is also a problem (because of an absence of inter-node adjustment); the acoustic signals have short ranges and maximum-scaling factors that cause a delay in communication. Therefore, we proposed Rotational Orbit-Based Inter Node Adjustment (ROBINA) with variant Path-Adjustment (PA-ROBINA) and Path Loss (PL-ROBINA) for IoUTs to achive reliable communication between the sender and sonobuoys. Additionally, the mathematical-based path loss model was discussed to cover the PL-ROBINA strategy. Extensive simulations were conducted with various realistic parameters and the results were compared with state-of-the-art routing protocols. Extensive simulations proved that the proposed routing scheme outperformed different realistic parameters; for example, packet transmission 45% increased with an average end-to-end delay of only 0.3% respectively. Furthermore, the transmission loss and path loss (measured in dB) were 25 and 46 dB, respectively, compared with other algorithms, for example, EBER^2^ 54%, WDFAD-BDR 54%, AEDG 49%, ASEGD 55%, AVH-AHH-VBF 54.5%, and TANVEER 39%, respectively. In addition, the individual parameters with ROBINA and TANVEER were also compared, in which ROBINA achieved a 98% packet transmission ratio compared with TANVEER, which was only 82%.

## 1. Introduction

The Internet of Underwater things (IoUTs) involves a group of sensor nodes that gather data from different sensors placed in the ocean’s depths to further transfer them towards the positioned devices at the external level through a network of midway nodes. The IoUTs consists of sensors and relay nodes that use acoustic mediums for communication. It can support various applications such as underwater tracking, climate exposure and recording, oceanographic information, pollution detection, underwater surveillance, navy defense systems, offshore exploration, and disaster prevention. As one-third of our population is surrounded by water and millions of people live within 100 km of the coastal area [1], the IoUTs will play a vital role.

State-of-the-art literature discourses the problems and limitations that interrupt the IoUTs’ effectiveness, including bandwidth limitation, high energy consumption, inadequate energy resources, efficient routing, and increased distribution cost [2]. Dynamic topology and water current are fundamental parameters for controlling the mobility factor inside the underwater field. The deployment of nodes before and after the experiment are quite different. Therefore, it is difficult to predict which node is inactive/alive/dead in shallow and deep water. Basically, the dead nodes are the gaps of nodes when the communication is near to the destination and is overburdened. When these nodes are found frequently in network size, the void nodes become void regions that isolate some part of the network. The main reason for this event is the absence of the candidate nodes that take responsibility for those nodes that are dying or are going to die. In addition, compared to Terrestrial Wireless Sensor Networks (TWSNs), identifying the localization of nodes is also difficult as radio signals do not work in underwater scenarios. For this reason, one of the best substitutes is the use of acoustics links. Moreover, energy in batteries or other means is limited and difficult to replace or re-charge in underwater scenarios.

In the literature, many routing schemes and algorithms have been reported to minimize the energy consumption of the nodes and identify dead nodes [3,4]. The dead nodes are those nodes that are not able to deliver the data to sonobuoys. For quick data delivery, many routing and flooding schemes use the shortest path and routing tables, angle-based flooding, the three-hop reliability model, watchman-based flooding, depth and location-based routing, pressure, and cluster/subnet-based approaches [5,6,7,8]. As nodes around the sonobuoys suffer, and the entire traffic load can die early, we propose the internodes adjustment mechanism for providing an alternative route by following the greedy forwarding approach to recover the data from the dead nodes. At the same time, Path Adjustment (PA) is perfomed by following the rotational orbit-based shape for alternative ways, collectively called Rotational Orbit-Based Inter-Node Adjustment (ROBINA).

Moreover, its two sub-variants: Path-Adjustment (PA-ROBINA) and Path Loss (PL-ROBINA), were proposed in this article. The ROBINA uses geographic energy-efficient data communication while PA-ROBINA provides proper path adjustment to bypass the dead nodes to discern the Immediate Available Forwarder Node (I_AFN_) after dead nodes. In addition, the PL-ROBINA uses the path loss transmission model to divert the path according to desired (I_AFN_). Moreover, the mode of operation is based upon the inter node adjustment mecahnisam. The relationship between rotation orbit and path selection is basically interlinked with each other. It is because of the rotation orbit-based pathway is used to find new node in the communication range. The path selection will automatically adjusted with the position of new finding node.

**Figure 1:**
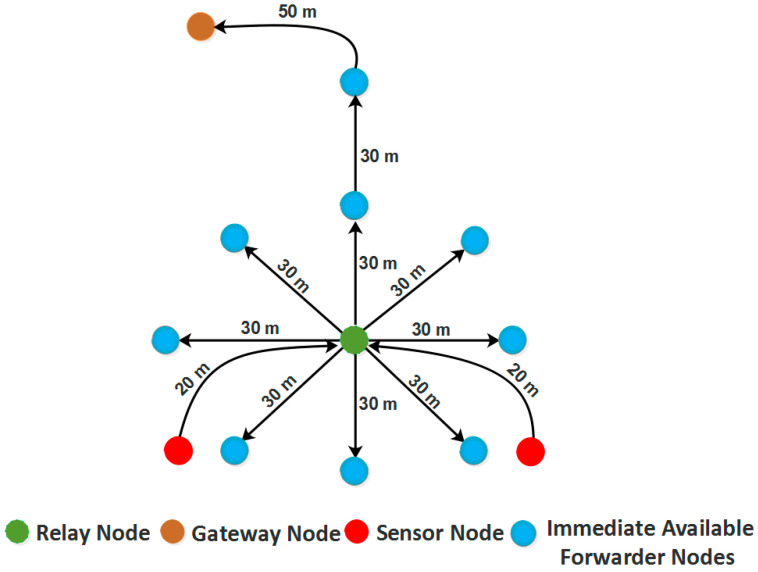
Mechanism of choosing the Immediate Available Forwarder Nodes (I_AFN_), adjusted by the proposed scheme ROBINA with the help of relay nodes and gateway nodes.

The mathematical-based formulas were introduced to create a path with a maximum rotation covering the main proposed approach, ROBINA. The possible model of this scheme is shown in Figure 1, where the occurrence of dead nodes is rescued through inter-nodes adjustment, and an alternate routing path is provided by a rotational orbit-based path as mentioned earlier.

Furthermore, the data received from dead nodes are covered by the internodes as these are responsible for transferring the data to sonobuoys. In case of data delivery failure to the internodes, the nearest sonobuoys automatically take the data using a first-come, first-served approach. In this regard, the PA-ROBINA and PL-ROBINA can provide a smooth communication path towards the sonobuoys and ensure that the data from the nodes (dead nodes) can be delivered to the destination.

In addition, the atomic shape-based data gathering routing protocol is already added to our contribution in the literature [9,10] for efficient delay and data gathering purposes. For this purpose, Autonomous Underwater Vehicles (AUV) are deployed along with member and gateway nodes [11]. Underwater AUVs are responsible for transferring the data to the derived destination without using internode adjustment; however, the long, unnecessary propagation delay occurs [12]. In addition, the extra consumption of energy and chances of packet dropping also make their impact [13]. Ultimately, the performance of the routing protocol is compromised. ROBINA takes this scenrion as an advantage for introducing inter-nodes adjustment between void holes rather than using AUVs.

Although the Potential Forwarder Node (PFN) can be replaced and substituted for inter-node adjustment for some reason, the establishment of the path, long routing cache, and criteria of selection of the PFN can be responsible for not outperforming in a well-disciplined manner. The dependency in EBER^2^ [14] (that uses the concept of PFN) also takes different transmissions for data delivery (which is not required; it is because we usually encourage more transmission in the vehicular network, ad hoc network, and for terrestrial networks). ROBINA does not use a different transmission; data delivery is performed through internode adjustment. Otherwise, packets will be dropped. Though depth is the most trustable factor in an underwater environment, it is not constant in oceans due to the dynamic nature of water surfaces. Therefore, line of sight direction, Zig-Zag path, and depth-based information followed by the shortest path cannot be performed well. As the underwater environmenmt is based upon the medium to large-scale networks, to cover this area with minimum energy consumption, there is a dire need to establish a path strategy that covers the area with minimum EC impact.

Extensive simulations were conducted, where the state-of-the-art routing protocols like AVH-AHH-VBF [9], ASEDG [11], AEDG [13], EBER^2^ [14], and WDFAD-DBR [15] were compared. It was demonstrated that the ROBINA outperforms affordable EC, minimum E2E delay, and high PDR. Our suggested method, ROBINA, and its co-variant PL-ROBINA employ Immediate Available Forwarder Nodes (I_AFN_). The basic function of using I_AFN_ rescues the routing path when it is established between two nodes. The relay nodes are randomly deployed inside the network that plays a part-and-parcel role to make the communication reliable and smoothly proceed the process of data packet delivery. From Figure 1, the (I_AFN_) near-relay nodes are available for communication as the subistiution for dead nodes. Relay nodes basically provide the information about these I_AFN_ with gateways for established communication between forwarder nodes and sink through the selected path.

Our Contributions. After introducing the idea underlines for rotation of path and its adjustment against various parameters, we proposed a rotational-based scheme that accordingly selects forwarder nodes in its routing path by ROBINA. We used Path Adjustment and Path Loss, called PA-ROBINA and PL-ROBINA, respectively for the adjustment and path loss mechanism. The PA-ROBINA is basically used for adjustment of the path based on the provided information by relay nodes and I_AFN_. As in IoUT, the nodes can only be remotely accessed, and unmanned effort is required for this environment to make the path consistent and reliable for acoustic IoT devices. We used the path loss function to detect the void nodes and rotate the path with the following ROBINA, called PL-ROBINA. The ROBINA and its co-variants work with I_AFN_, which is the core mechanism of internodes adjustment. The criteria to choose the I_AFN_ was provided, while the mathematical model was also produced to select the optimal I_AFN_ with the help of Urick’s Model and Thorp’s Formula. The contributions can be summarized as follows:

■ We presented the ROBINA for inter-node modifications in all possible directions following path rotations until all nodes are designated for communication and are within the radius of the rotated path.
■ We also analyzed the relay nodes quantity that is selected under the criteria of path rotation and adjusted its position by PA-ROBINA. It also uses the I_AFN_ function and Parity bit-based flags to choose nodes that are closest to the destinations.
■ We opted for and reformulated the ambient noise-reduction solution using the Urick’s Model for acoustic signals, and absorption loss factor by Thorp’s Formula, that modeled continuous Power Spectral Density (PSD) and Colored Gaussian Statistics (CGS). All these work for PL-ROBINA.
■ We evaluated the first rotational orbit-based proposed scheme’s performance by comparing it with state-of-the-art benchmark schemes under different parameters of transmission loss, throughput, number of dead nodes, and etc.

The rest of the work is organized as follows. We reviewed state-of-the-art literature in Section 2 and provided the problem formulation in Section 3. Furthermore, Section 4 and Section 5 present the proposed schemes and their sub-sections. The path loss channel model is described in Section 6. Finally, the simulation results and conclusion are provided in Section 7 and Section 8, respectively.

## 2. Literature Review

Recently, the demand for the deployment of sensor networks in the underwater environment has gained significant attention in the networking domain with unique features and continuous sensing movement.

A protocol for the IoUTs, namely Energy-Balanced Efficient and Reliable Routing (EBER^2^) is discussed in [14] to balance energy and achieve reliability. EBER^2^ deliberates enduring significance and the number of PFN transmission choices divided among the power levels to enhance energy effectiveness. The protocol allows forwarders to adaptively control their communication according to the utmost node in the neighbor list of the network. To reduce the duplication of packets, residual energy, PFNs, and their depths are compared among the neighbors. Moreover, two sinks are deployed at high-density traffic areas to reduce network latency. The study results show that EBER^2^ has lowered energy consumption and identical packets with low packet delivery ratios compared with the Weighting Depth and Forwarding Area Division-Depth Based Routing (WDFAD-DBR) protocol [15]. The WDFAD-DBR mechanism considers the depth of the next forwarding node, through which it avoids void holes. Multiple IoUTs schemes have been designed to prevent voids holes, including AVH-AHH-VBF and SM-AHH-VBF [9]. These schemes’ main concern is to raise the lifespan of the sensor network system with low energy consumption. The study results show that the proposed methods outperform high energy consumption with an end-to-end interruption in packet transfer, average packet delivery ratio, and average propagation distance compared with the baseline solution.

Another issue is imbalance of energy consumption that degrades the overall performance of sensor-based networks with high data-traffic flow between intermediate nodes [3]. Many researchers have worked for efficient energy consumption and data-traffic distribution to reduce the intermediate node’s packet forwarding ratio. For this purpose, an Energy Grade (EG) and load-balance-distributed corona were proposed in [10]. A corona-deprived EG and a corona deprived of Depth Adjustment (DA)-enabled schemes are proposed to allocate the flow of data traffic nodes for effective energy distribution. The dynamic alteration of the communication range in the initial method supports the reduction of data load. Both EG and DA work to allocate data traffic for equal distribution of energy.

IoUTs face serious packet delivery problems, packet loss, and collisions because of the solid placement of the sensing nodes in severe underwater environments. The retransmission of the packets causes a lot of energy consumption and interruption, and delays in packet delivery. A framework was proposed in [4] to minimize energy loss, maximize throughput, and improve the network’s lifetime. A three-dimensional 3D acoustic scenario [5] usually works with a distributed sink that is not static in network stability, packet loss analysis, and lifetime. Furthermore, the proposed framework was compared with prevailing techniques like Mobicast and iAMCTD protocols that pointed out node compactness in the network with an adaptation of varying depth consequences and DA in low energy consumption reduces retransmission and improved throughput [8]. The proposed framework delivers an improved performance for real-time interruption-based applications over the current mechanisms.

The major challenge of the IoUTs is sending data towards the sink stations; due to the continuous movement of the nodes, it becomes difficult to transfer data. In order to overcome this problem, we make the orbit-based routing path in our proposed scheme with supporting thories listed in [16,17] and Opportunistic Routing Protocols (ORPs) [18,19] are examined to expand performance with a dynamic selection of one best forwarding device from the other. Location-Based Opportunistic Routing Protocols (LBORPs) are recognized and identified to perform better using knowledge for dynamic selection of forwarder devices through their location and route message packets to the receiver [7]. The functioning of location-focused routing protocols is studied to perform well in IoUTs atmospheres. Hence, these location-based protocols decrease performance in IoUTs networks due to communication ejects or holes (sometimes called void holes and region) [5]. The researchers discussed the working of LBORPs communication voids in IoUTs along with their problems and disadvantages. The first LBORP is VBF and the second one is HH-VBF, which are analyzed where VBF establishes a simulated vector pipe for routing among source and receiver sink nodes, only those in the virtual vector. Whereas, in the HH-VBF protocol, each node uses different virtual pipes and during each time of transmission the direction of the virtual pipe changes. A comparison of both VBF and HH VBF protocols was performed to analyze the performance of both protocols, and the networks consequently received better performance without communication void holes. Routing issues and challenges related to the IoUTs were identified to assist the scholars in developing and deploying efficient IoUTs routing protocols with multiple communication holes. Further the estimated distance based approaches are discussed [20,21] in which the routing path and its calculation from sink.

Another routing technique called TANVEER, with its three sub-sections, LBA-TANVEER, DPD-TANVEER, and BIN-TANVEER, experimented with IoUTs [22]. These schemes are based on triangular calculations to bypass empty regions and follow the smooth route towards destinations. The author used a straightforward approach to mitigate the blank nodes inside the routing path. The experimental results suggested that the TANVEER and BIN-TANVEER secured satisfied marks against a fraction of empty regions.

## 3. Problem Formulations

Two out of four routing techniques proposed different routing paths that directly connect the source and sinks through intermediate, relay, and forwarder nodes. The probability of packet losses, node failure, energy tax (extra), and inefficient data gathering in these schemes is common. Furthermore, the direct path through mediators’ nodes is an old technique in which EBER^2^, WDFAD-DBR, and AVH-AHH-VBF are inline. With reference to their simulation, unsatisfactory results are achieved. Moreover, the AEDG and ASEDG both introduce the atomic shape path for relay nodes and sinks. Still, it is not rotational, so there is a need to design a routing path that is shortest in length and covers the parameters mentioned above. In our proposed work, there was some consideration because of our focus on the avoidance of void nodes by introducing inter-node adjustment and providing alternative paths via rotational orbit-based shapes (as orbits are much denser then atomic and elliptical shapes and cover a larger area [11,13]). The first consideration is: (i) every node has its localization information, (ii) symmetric communication was assumed between two nodes, for example, ‘*i*’ and ‘*j*’, (iii) sinks can receive multiple buckets at the same time, (iv) the vertical direction in water was mostly not considered, but due to water current, the horizontal direction was considered, (v) dead nodes were near the sinks called Sonobuoys Neighboring Nodes (SNNs), and its orbit path was considered for these SNNs when introducing inter-node adjustment (which is the proposed scheme), and (vi) if the SNNs zone is closer to the sinks, then the internode has sent the data directly without taking an alternative path to improve network throughput [19]. The major contribution of our work is as follows.

Our contribution in this regard was to make the path officially rotational (based on inter-nodes adjustment) and have orbits shape, not atomic and elliptical [11]. It is interesting to know about atomic that its particle is itself surrounded by rotation. However, the atomic particles that move around its central point also have the shape of orbits. The major advantage of the orbit-based path covers much area as compared to atomic path and elliptical. The two-way motion described in the literature according to *Heisenberg*’*s Principle* [16] is possible, in which they state that *“every particle have two-way motion, first itself and other around the material in which they occupied”* [20].

In addition, the orbit is larger than a circle, and quite similar to an elliptical shape; on the contrary, the atomic path is also under the umbrella of eclipse shapes. From this reference, we can say that the ancestor of these shapes (including the atomic shape path used in ASEDG [13]) of the orbit has more advantages and result-oriented features in all relative parameters.

Furthermore, the area covered by the atomic path is not much greater than the orbit path mentioned in ‘*Bernoulli*’*s Equation*’ [17]. It states that the area covered by its volume is always directly postponed to its ‘*pressure and performance.*’

## 4. The Rotational Orbit Based Inter Node Adjustment (ROBINA)

**Figure 2:**
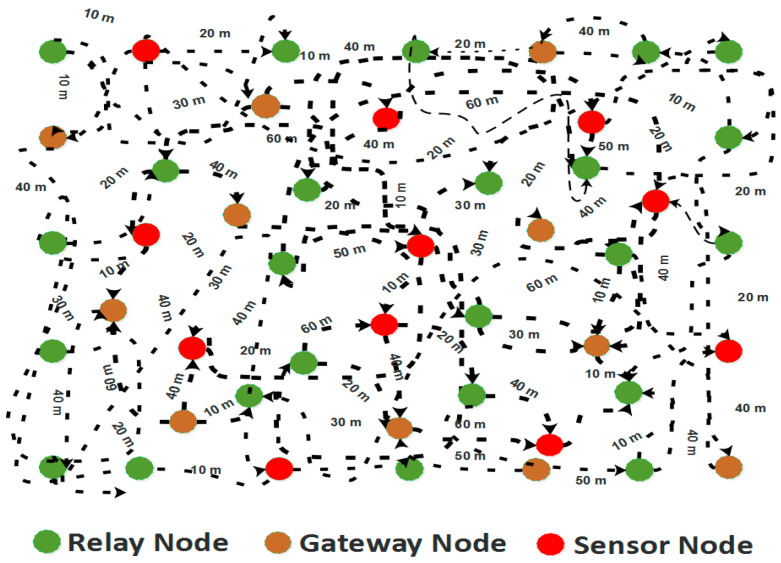
Nodes are randomly deployed in the IoUTs scenario without applying the ROBINA algorithm (especially when the path is not established).

The ROBINA takes the available number of nodes and its neighboring intermediate forwarding node around the sinks for reliable data transmission. Firstly, the sensor, relay, and gateway nodes are nominated and randomly deployed without the ROBINA, as shown in Figure 2. All these nodes are deployed as anchored positions, and take data information towards the sinks. In order to avoid a collision, it is assumed that one node can carry and transfer the data one packet at a time [23,24,25,26]. Due to the increased traffic volume, the empty void nodes form void regions. At that location, the internode adjusts and transmits a beacon message requesting an alternate. An alternate path based on rotational orbits is established around the SNNs. Its radius must include both the node’s void dead area and the set of neighboring nodes. It consists of multiple nodes that ensure the recovery of data with higher priority. The following mathematical expression is used to adjust internodes around SNNs in Equation (1).

(1) $$A\;\left(AdjNi\right)=\frac{E\;\left(Ni\right)}{\left(\;SNN_{range}*N+f(Ni\right)}$$

where *f*(*Ni*) = {1|0} (=0 for a dead state, or =1 for an alive state)

From Equation (1): the *A* (*AdjNi*) is the selected area for the proposed scheme in the network that firstly calculates the *E*(*Ni*) energy of the node and the range of ($SNN_{range})$ where *f(Ni)* = {1|0} (=0 for a dead state, or =1 for an alive state). In the equation described above, we take a scenario in which node ‘*i*’ sends data to node ‘*j*’ with energy ‘*E*’; first, it explores the ‘*Ns*’ towards the ‘*Sn*’, then, for all nodes that wait for Packet ACKnowledgment (*P*_ACK_), takes the time (t) in such a way that *N_ij_* = {*n_i_* ∈ *n_j_ ^ E_ij_:* ∀ *AdjNi ∗ SNNs + Dn*}, for orbit pathTp = $\sum_{n=i}^{j}\left(Nij*SNNs\right),\;$then P_ACK_ in total time for path establishment counts the set of neighboring nodes with sink [27] as mentioned in Equation (2) with Selection Operation (*Sel_op_*).

(2) $$Sel_{op}=\frac{Ns-Dn}{Sn}$$

The following equation ensures that the *P*_ACK_ from Node ‘*i*’ to ‘*j*’ with ‘*E*’ is as expected in Equation (3), where *P*_ACK_ is discussed in detail.

(3) $$P_{\mathrm{ACK}}\;\left(N\right)=\prod_{j=0}^{d=1}\forall \;SNNs\;\left(Dn\right)\subseteq Sn$$

Additionally, in the following section with Algorithm 1, the procedure of (I_AFN_) for forwarding the Beacon Message (BM) is discussed. In Algorithm 2, the detailed description of data delivery using a path adjustment mechanism is discussed. The PA-ROBINA works with internode adjustment as I_AFN_; while setting the internodes between the void nodes, the path will automatically adjust at that region and rescue the dead nodes. For the adjustment of the region of the path, the range of *SNNs* plays a role in determining which path is closer to sonobuoys so that it is easier for the path-based nodes like internodes to send the acknowledgment of the nodes that are not alive [28,29,30,31,32,33].

**Figure 3:**
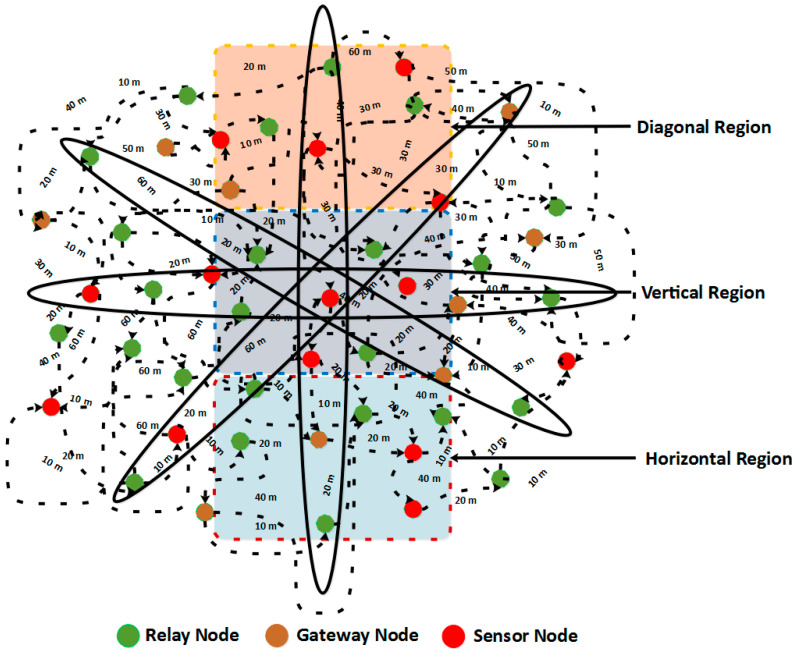
Acoustic Scenario with Path Adjustment of ROBINA.

**Figure 4:**
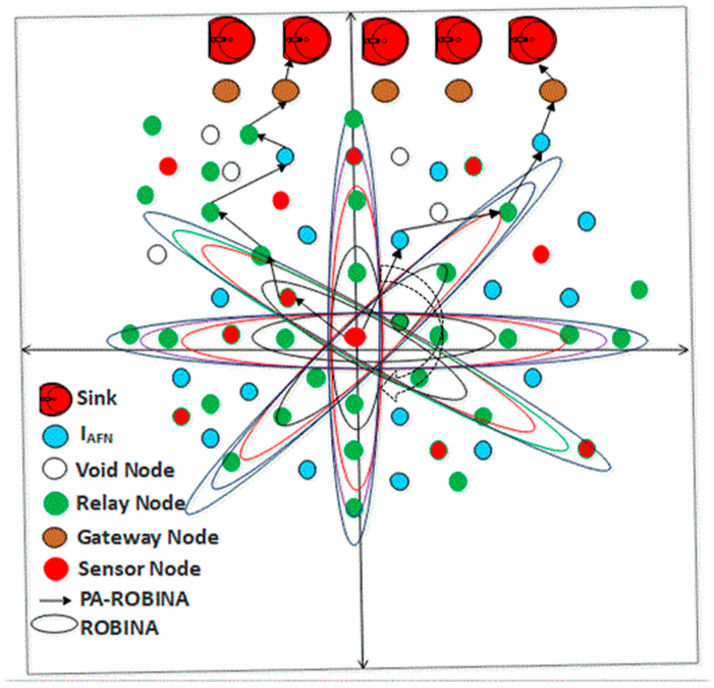
The woring of PA-ROBINA in acoustic scenario.

Moreover, the working of ROBINA for the adjustment of internode and its PA-ROBINA for setting the rotational shortest path are described in Algorithms 1 and 2, respectively. In Figure 3, the overall working of ROBINA is explained with its acoustic scenario. Usually, the scenario Underwater consists of relay nodes, sensor nodes, and gateway nodes. The relay nodes are considered as the immediate medium to pass the communication from source to destination. Gateways are present to receive data packets from relay nodes, which arepassed through the desired destination. The whole scenario is followed by an intentionally rotating path (from left to right) that is orbital in shape. The increasing trend of every orbit shape presents the refeclection of the rotating path where needed. The inside circle shows the overall rotation of the path to avoid void holes and deploy the inter-node adjustment near the SNNs. The ‘*oval*’ legend in Figure 4 shows the working of the orbital routing path while the ‘*arrow*’ denotes the straight forward path adjustment of ROBINA.

Further, the nodes closer to the destination take enough burden from the whole network communication and die early, which relay as void nodes (white in color). When these nodes are found frequently in network size, they become void regions isolated to some part of the network too. The main reason for this event is the absence of the candidate nodes responsible for those nodes that die or near death. From this reference, we introduce the idea of inter-node adjustment and formulate their path accordingly. Usually, in underwater situations, the path is horizontal, vertical, elliptical, atomic shaped, vector and depth-oriented, height and angle oriented, or etc. Due to the non-rotatable behavior of the path, all these schemes are not well performed. Therefore, a rotational-based path with an internode adjustment mechanism performs well when the area of interest is dense and shallow. The novel orbit-based path rotates with the adjacency of relay nodes, including some inter-nodes adjustment (where needed), making the communication secure and reliable.

## 5. Description of Algorithms 1 and 2

In this section, Algorithms 1 and 2 for inter-node and path adjustment are described in detail; first, the *BM* are decided for forwarding and receiving acknowledgment for relay and sensor nodes, in which each ‘*BM*’ contains next node’s information with its correspondent coordinates to determine the location and distance of nodes. All this information is simultaneously updating in the routing table as [28,29,30].

**Figure 5:**
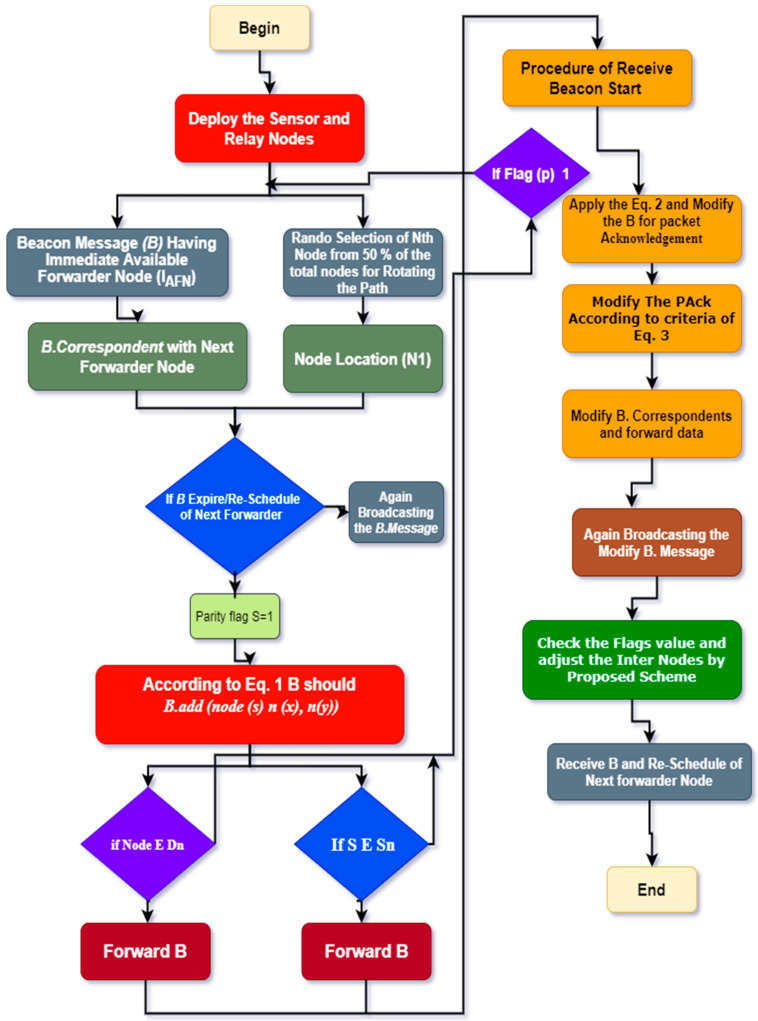
Workflow of the proposed ROBINA scheme.

**Figure 6:**
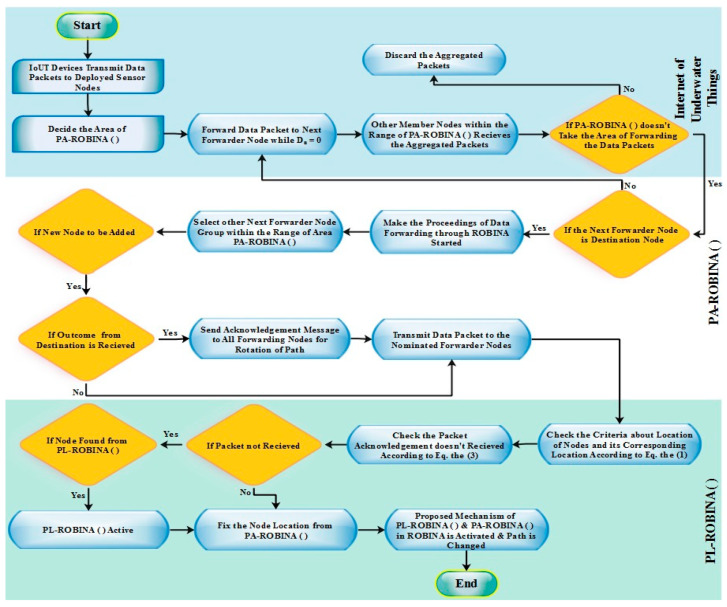
Proposed working mechanism of PA-ROBINA and PL-ROBINA.

To add the next node, the mechanism of parity flag is introduced if it is 1, then forwarder nodes with all their adjacency are added to set out the packet orientation, and ignored if it is 0. The path should be modified (due to the rotational mechanism technique used in ROBINA) if the *P*_ACK_ does not receive from adjacent nodes as described in Equation (2). The modified *P*_ACK_ is used to set down the path in the desired direction, as mentioned in Algorithm 1. The delivery of data using path adjustment only considers the next forwarder nodes and their re-scheduling. If the distance of nodes does not have a minimum value for sink nodes and the next forwarder nodes, then adjacent nodes will be put up as the next forwarder node and all other nodes are rescheduled by avoiding the void nodes to make the path clear for the destination. In Figure 3, the whole network is divided into three sections: a horizontal region, a vertical region, and a diagonal region, respectively. According to our proposed techniques, three types of nodes make communication possible: relay nodes, sensor nodes, and gateways nodes. The PA-ROBINA mechanism is accordingly adjusted with the help of relay nodes. If void nodes want to become network members, the inter-node adjustment mechanism is activated, and the path is adjusted according to inter-node placement. This mechanism is described with distance in Figure 4 In addition, Algorithm 2 is discussed with data delivery using the PA of ROBINA. For this, the area of path adjustment via relay and inter-nodes is decided. Suppose nodes are present within the path’s defined area. In that case, the path is rescheduled according to the role of inter-node adjustment, and data packets are forwarded to the destination’s desired direction. In Table 1, all the acronyms with their descriptions are listed. In addition, the flowchart of ROBINA and its variants like PA-ROBINA and PL-ROBINA are described in Figure 5 and Figure 6, respectively.
**Algorithm 1: The ROBINA.**  B: Beacon message having next node  Dn: Set of Dead Nodes  S: Sonobuoys  S_n_: Set of Nodes  Parity Flag Values: 0 and 1 (Used to Check Void Nodes)  X. Y. Z: Coordinates of nodes  Adj N_i_: Adjacent Nodes  Procedure **FORWARD BEACON** (Sonobuoys (S_n_), Node)  **If B** expires, **then**//*Using B in Underwater is like ‘Hello Packet’ having the address of the next node*  B. correspondent ← Node location (N_l_)//*correspondent having all possible coordinates of Nodes*  **if** node ∈ D_n_
**then**//*check for dead nodes*            **for** S ∈S_n_ node, **do**  **if** parity flag (s) = 1 **then**               B.add (node (s) ← n (x), n(y))  *A (Adj_Ni_) =* $\frac{E\;\left(Ni\right)}{\left(\;SNN_{range}*N\right)+1}$//*adjacency nodes are decided using Equation (1).*  Flag (p) ← 0//Receive Partiy Value  **end if**  **end for**  **end if**
  Forward **B**  **End if**  **End Procedure**  Procedure **RECEIVE BEACON** (Node, B)  **If B** ← S_n_            Modify **B**. correspondent then            **For** $Sel_{op}$ = $\frac{Ns-Dn}{Sn}$//*using*
*Equation (2)*  P_ACK_ = *A (Adj_Ni_) ∗ SNNs*  ***Else***
  *Modify P_ACK_ (N)*_=_ $\prod_{j=0}^{d=1}\forall \;SNNs\;\left(Dn\right)\subseteq Sn$//*using*
*Equation (3)*  ***if*** $flag{\;}_{p}^{j=0}\;\leftarrow 0$               Receive **B**  **end if**  **End For**  ***End IF***  ***End Procedure***


**Algorithm 2: Data Delivery Using PA-ROBINA.**
  B: Beacon message having next node  Dn: Number of Dead Nodes  S_n_: Set of Nodes  Ns: Set of Neighboring nodes  Adj N_i_: Adjacent Nodes **Procedure** Area of Path Adjustment (*A*) **IF** |D_n_| = 0 **then**  Forward data packets ()  **Else**   D_n_ ← takes the next forwarder node (n)    **If** |D_n_| = 0 **then**      **for** *A (Adj_Ni_) = N_s_* − D_n_ do      Forward data ←S    **Else**      Re-schedule of next forwarder node      **Proposed_scheme ()**    **end if**       **end for** **End IF**  **End Procedure**

## 6. Path Loss Channel Model

Rotational Orbit-Based Inter-Node Adjustment (ROBINA) and its Co-variant Path-Adjustment (PA-ROBINA) work for Path-Loss (PL-ROBINA) respectively. Underwater, the acoustic channels are determined by the operating frequency as well as the distance. Here, the operating frequency is only considered between receiver and transmitter as mentioned in [31,32,33]. Underwater path loss dependability is quite different in practice in Terrestrial Wireless Sensor Networks (TWSNs). Path Loss (PL) is determined only by the distance between communication nodes in Radio Frequency (RF) [34] between two communication nodes; particularly, the acoustic signal undergoes a frequency ‘offshore for a distance ‘*l*’. From the Urick’s Model enlisted in [34], the overall PL is denoted by ‘*A*(*l,f*)’; finally, we have to modify it according to the scenario.

(4) $$A\left(l,f\right)=l^{j}{\left(l\left(f\right)\right)}^{\frac{d}{1000}}$$

The ‘*j*’ represents the circulating factor and describes acoustic signal propagation geometry. Typically, the practical value for ‘*K*’ in the literature is 1.5. The acoustic pressure into heat for the absorption loss factor α(f) is modeled in Equation (4).

According to Thorp’s formula [35], usually ‘*f*’ measured is *KHz* as following in Equation (5):

(5) $$A\left(f\right)=10\log\left(\alpha \left(f\right)\right)\;\mathrm{dB}/\mathrm{km}\;=\frac{0.11f^{2}}{1+f^{2}}+\frac{40f^{2}}{4100+f^{2}}+2.75\times 10^{-4}f^{2}+0.003\;$$

According to [36], there are some main backers defined to the ambient noise like thermal noise *N_th_ (f)*, turbulence *N_th_ (t)*, shipping and human activities *N_s_ (f)*, and wind-driven wave *N_w_ (f)*, respectively. The continuous Power Spectral Density (PSD) and Colored Gaussian Statistics (CGS) are the major supportive parameters for modeling these equations. Furthermore, the PSD formulae of all these four types of noises with taking frequency ‘*f*’ (measured in *kHz*) are given empirically below in Equation (6):

(6) $$10\log\left(N_{t}\left(f\right)\right)=19-40\log\left(f\right)\;10\log\left(N_{s}\left(f\right)\right)=50+30\left(s-0.6\right)+28\log\left(f\right)-80\log\left(f+0.04\right)\;10\log\left(N_{w}\left(f\right)\right)=60+7.5w^{\frac{1}{2}}+30\log\left(f\right)-50\log\left(f+0.5\right)\;10\log\left(N_{th}\left(f\right)\right)=-18+30\log\left(f\right)$$

Wheres ∈ [0, 1] is defined as 0 in legal shipping activity, ‘*f*’ in high-level shipment movement, and ‘w’ is the storm velocity measure in m/s; the ‘*w*’ is also utilized by wind-driven waves that cause surface motion to be captured. Furthermore, the total ambient noise’ *N* (*f*)’ is calculated as in Equation (7):

(7) $$N\left(f\right)=N_{t}\left(f\right)+N_{s}\left(f\right)+N_{w}\left(f\right)+N_{th}\left(f\right)$$

The SNR of an inbound acoustic sound of frequency ‘fi’ transmitted over a distance’ *d_j_*, to node ‘*n*’ is represented as [36].

(8) $$SNR\left(d_{j,n},f_{i}\right)=\frac{{\left|a_{j,n}^{i}\right|}^{2}P_{j,n}^{i}}{A\left(d_{j,n},f_{i}\right)N\left(f_{i}\right)B_{S}}$$

where:

(1) *P^i^_j,n_* shows the power transmission between two nodes.
(2) *N(f_i_)* is the noise power spectral density.
(3) ‘*B_s_*’ denotes bandwidth on the receiver side.

In multiple scenarios in Underwater, we only assume the fading ratio of a particular channel between two nodes on a limited scale owing to multipath |*d^i^_j,n_*|. Here, we assume |*a^i^_j,n_*| follows the same work as described in [37,38].

Optimum Acoustic Carrier for Data Transmits Model

The suggested protocol is based on mathematical models that show the smallest amount of transmission that is normally compulsory for data rate per connection. Assume *P^i^_j,n_* is used for transmitting packets from node ‘*j*’ to node ‘*n*’ node subcarrier ‘*i*’. The goal is to determine the lowest feasible transmit power over a set of ‘*x*’ subcarriers while maintaining the required transmission rate, indicated by ‘*R*_0_’ between the nodes ‘*j*’ and ‘*n*’. The following is a summary of the entire scenario:

(9) $$\min\left\{\overset{x}{\underset{i=1}{\sum }}P_{j,n}^{i}\right\},\;s.t.B_{s}\overset{x}{\underset{i=1}{\sum }}\log_{2}\left[1+SNR\left(d_{j,n},f_{i}\right)\right]\;\;\;\;\geq R_{0}\;and\;P_{j,n}^{i}\geq 0\;\forall \;i,j,n$$

*SNR* (*d_j,n_, f_i_*) is derived from Equation (8), and ‘*x*’ is the number of sub-channels utilized by node ‘*j*’ to send data to node ‘*n*’. It is worth noting that it is just like the convex function that is used for the objective function.

The function of $B_{s}\sum_{i=1}^{x}\log_{2}\left[1+SNR\left(d_{j,n},f_{i}\right)\right]\;$is in terms of *P^i^_j._* All these details are described in Equation (9) to minimize the cost function [39]. We differentiated Equation (10) with some derivatives by substitution, in which we used ‘λ’ as a long-range multiplier.

(10) $$L\left\{{\left(P_{j,n}^{i}\right)}_{i=1}^{x},\lambda \right\}=\overset{x}{\underset{i=1}{\sum }}P_{j,n}^{i}-\lambda \left\{B_{s}\overset{x}{\underset{i=1}{\sum }}\log_{2}\left[1+\frac{{\left|a_{j,n}^{i}\right|}^{2}P_{j,n}^{i}}{A\left(d_{j,n},f_{i}\right)N\left(f_{i}\right)B_{S}}\right]-R_{0}\right\}$$

(11) $$\frac{\delta L}{\delta P_{j,n}^{m}}=\frac{1-\frac{\lambda }{ln2}\left[\frac{{\left|a_{j,n}^{m}\right|}^{2}}{A\left(d_{j,n},f_{m}\right)N\left(f_{m}\right)}\right]}{1+\left[\frac{{\left|a_{j,n}^{m}\right|}^{2}P_{j,n}^{m}}{A\left(d_{j,n},f_{m}\right)N\left(f_{m}\right)B_{S}}\right]}=0\;$$

Here we can elaborate Equation (11) for *P^m^_j,n_*, as:

(12) $$1-\frac{\lambda }{ln2}\left[\frac{{\left|a_{j,n}^{m}\right|}^{2}}{A\left(d_{j,n},f_{m}\right)N\left(f_{m}\right)}\right]=1+\left[\frac{{\left|a_{j,n}^{m}\right|}^{2}P_{j,n}^{m}}{A\left(d_{j,n},f_{m}\right)N\left(f_{m}\right)B_{S}}\right]\;\frac{ln2A\left(d_{j,n},f_{m}\right)N\left(f_{m}\right)-\lambda {\left|a_{j,n}^{m}\right|}^{2}}{ln2A\left(d_{j,n},f_{m}\right)N\left(f_{m}\right)}=\frac{A\left(d_{j,n},f_{m}\right)N\left(f_{m}\right)B_{S}-{\left|a_{j,n}^{m}\right|}^{2}P_{j,n}^{m}}{A\left(d_{j,n},f_{m}\right)N\left(f_{m}\right)B_{S}}$$

Since in cross multiplication ‘$P_{j,n}^{m}$’ will disappear and become a free variable, and we can assign any value to it.

(13) $$B_{S}\left[ln2A\left(d_{j,n},f_{m}\right)N\left(f_{m}\right)-\lambda {\left|a_{j,n}^{m}\right|}^{2}\right]=ln2\;\left[A\left(d_{j,n},f_{m}\right)N\left(f_{m}\right)B_{S}-P_{j,n}^{m}\right]\;P_{j,n}^{m}=B_{S}\frac{[\lambda {\left|a_{j,n}^{m}\right|}^{2}-ln2A\left(d_{j,n},f_{m}\right)N\left(f_{m}\right)}{ln2{\left|a_{j,n}^{m}\right|}^{2}}$$

We obtain:

(14) $$P_{j,n}^{m}=B_{s}{\left[\frac{\lambda }{ln2}-\frac{A\left(d_{j,n},f_{m}\right)N\left(f_{m}\right)}{{\left|a_{j,n}^{m}\right|}^{2}}\right]}^{+},\;\forall \;m=1,2,\ldots ,x\;$$

where ‘+’ means to have a source of projections taking non-negative numbers.

(15) $$\frac{\delta L}{\delta \lambda }=0\;\Rightarrow \;B_{s}\overset{x}{\underset{i=1}{\sum }}\log_{2}\left[1+\frac{{\left|a_{j,n}^{i}\right|}^{2}P_{j,n}^{i}}{A\left(d_{j,n},f_{i}\right)N\left(f_{i}\right)B_{S}}\right]=R_{0}$$

We obtain from Equation (14), borrow the Equation (15) values, and perform simple procedures.

(16) $$\lambda =\left[\frac{part_{1}}{part_{2}}\right]\left(ln2\right);\;where\;part_{1}=2^{\frac{R_{0}}{xB_{s}}}\;and\;part_{2}={\left[\overset{x}{\underset{i=1}{\prod }}\frac{{\left|a_{j,n}^{i}\right|}^{2}}{A\left(d_{j,n},f_{i}\right)N\left(f_{i}\right)}\right]}^{1/x}$$

Solving for the minimum power $P_{j,n}^{m}$ we obtain: $P_{j,n}^{m}=B_{S}{\left(\frac{part_{1}}{part_{2}}-\frac{part_{3}}{part_{4}}\right)}^{+}$; *where* $part_{3}=A\left(d_{j,n},f_{m}\right)N\left(f_{m}\right)$ *and* $part_{4}={\left|a_{j,n}^{i}\right|}^{2}$.

Therefore, the number of sub-channel ‘*x*’ is used by node ‘*j*’ to transmit data to node ‘*n*’.

## 7. Simulation Parameters

The extensive experimental setup connected 700 nodes that start from 50 until it reached its limits, i.e., 700 nodes. A 3D environment with 1600 × 1000 × 1000 dimensional area was used with acoustic link 1500 m/s and 2 kh bandwidth. The Beacon Message size was around 52 bits. All the other parameters like packet size data rates, transmission range, and initial energy were the same as mentioned in [11] and are listed in Table 1. Usually, vertical movement of nodes is not considered underwater; additionally, 2 m/s of horizontal movement was taken into account. Networks simulated NS-2 with version 2.35, along with a dedicated AquaSim framework used for simulations. Table 2 shows the list of simulation values.

### 7.1. Performing Assessment

In this section, we evaluate the performance of ROBINA against different state-of-the-art routing protocols like EBER^2^ [14], AEDG [13], ASEDG [11], WDFAD-DBR [15], AVH-AHH-VBF [9], and TANVEER [22]. The parameters were average end-to-end delay, network lifetime, total energy consumption, and packet success ratio. Finally, the overall performance trade-offs of under-observed and some general algorithms in this domain are listed in Table 3.

### 7.2. Analysis of Number of Dead Nodes, Packet Transmitted, and Average End to End Delays

**Figure 7:**
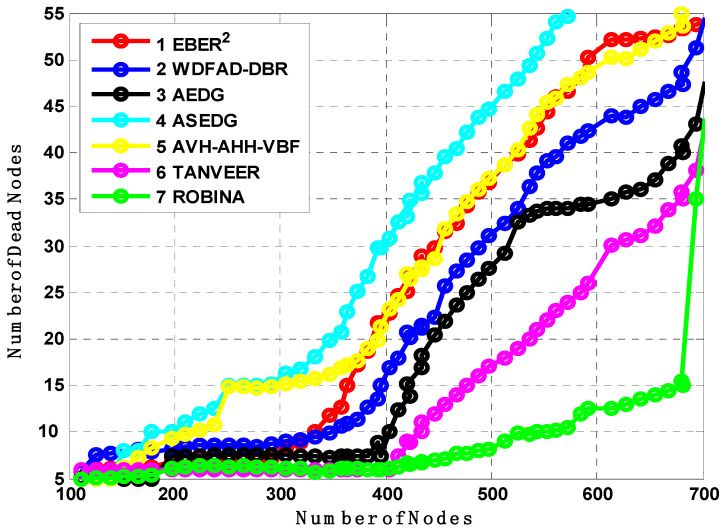
Analysis of the number of dead nodes with baseline schemes.

**Figure 8:**
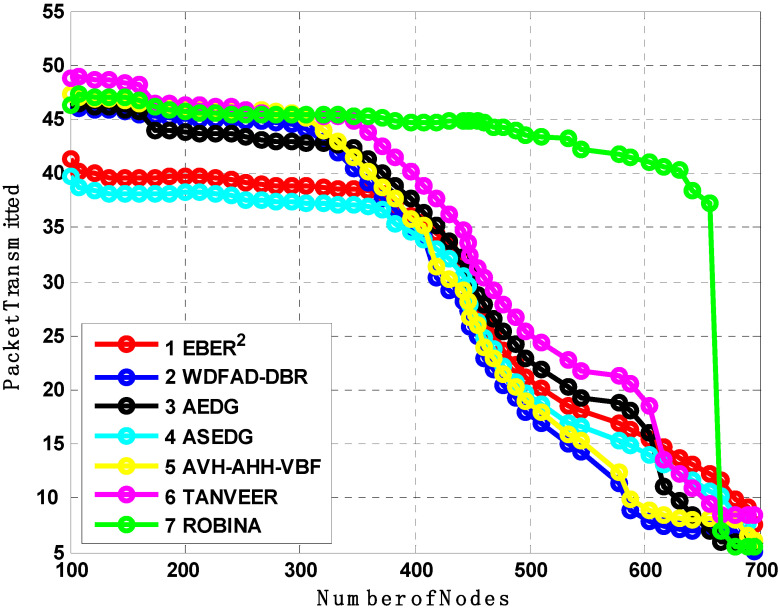
Analysis of Packet transmission with baseline schemes.

**Figure 9:**
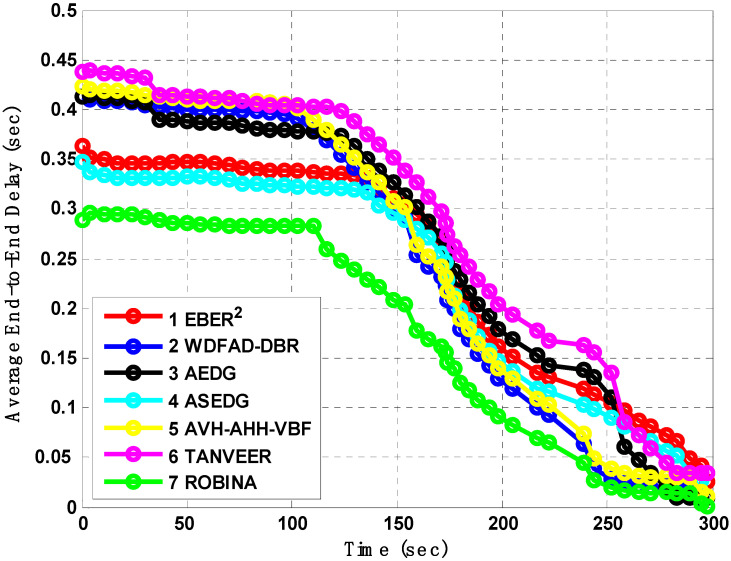
Analysis of End-to-End average delay with baseline schemes.

Figure 7, Figure 8 and Figure 9 evaluate all benchmark schemes against the proposed scheme ROBINA. For example, EBER^2^ is a crook with balance energy and the number of PFN divided among the power level to enhance energy effectiveness. The criteria by which EBER^2^ selects and deploys PFN is not energy efficient; this is why a large number of nodes (when it is experimental with ROBINA) is nearly boosted up when only the number of nodes is around 400. As the number of nodes increased by 450, it continuously followed the same behavior towards an increasing peak. The same trend is followed by WBFAD-DBR, which chooses the depth of the next forwarder node to avoid void holes. Since mobility is used with AVH-AHH-VBFA, it successfully raises the lifespan of the network with maximum throughput. Therefore, this study showed an outperformance of EBER^2^ and WDFAD-DBR, respectively when compared with ROBINA as the baseline scheme. It was discovered that AVH-AHH-VBF (due to sinking mobility), WDFAD-DBR (due to using depth divisions with traditional DBR), and EBER^2^ (due to using (PFN) in its nodes criteria selection) all disregard the following things. (i) criteria towards adjustment of inter-nodes (by any means, either in depth-divisions or PFN and next forwarder nodes). (ii) Selection of shortest path (mentioned in EBER^2^ and WDFAD-DBR and the hop-base path followed by AVH-AHH-AVBF) instead of the reliable connected path between nodes. (iii) Proof of work (by some mathematical analysis, formula, or some linear equations) is not followed by any existing state-of-the-art schemes. (iv) The path adjustment and path loss (if any) between two connected nodes are not discussed; likewise, all these parameters are covered with PA-ROBINA and PL-ROBINA. Therefore, the total number of dead nodes for EBER^2^, AVH-AHH-VBF, and WDFAD-DBR are 53, 50, and 52, respectively, when the total number of nodes is experimented in network 700. On the contrary, both AEDG and ASEDG have established the atomic-based path that rotates around the sink and an efficient delay and data-gathering approach that is further divided into atomic-shaped trajectory elliptical for autonomous underwater vehicles (AUVs). Although the path is atomic-shaped and has an elliptical fashion, it is not rotatable. A rotation is missing in AEDG and ASEDG; therefore, most of the relay nodes are not considered, and this is why it is only for 45 nodes against ten iterations, which is comparatively less, and against EBER^2^, AVH-AHH-VBF, and WDFAD-DBR, respectively, but much higher than ROBINA, which has only 39 dead nodes. TANVEER and ROBINA both show the most interesting results regarding dead nodes. As TANVEER and ROBINA are working to avoid void nodes, even their sub-variants are also working to support the main mechanism. Due to its triangular approach, TANVEER is partially rotated, and ROBINA is a fully rotated orbit-based approach up to 400 nodes; both have the same fashion loss of any nodes as dead. The increasing trend seems to be followed by TANVEER due to its angle cone; as data is forwarding repeatedly and measuring its cone, the rest of the nodes are compiled with energy drastically. Consequently, we can say that as a greater number of nodes appear as dead nodes, ultimately, the performance of packet transmission decreases. Simultaneously, across the board, EBER^2^, ASEDG, and ASEDG have the same packet transmission ratio, approximately which is 40%. Other schemes, including ROBINA, TANVEER, AEDG, WDFAD-DBR, and AVH-AHH-VBF, have 42%, 43%, 41%, 40%, and 42%, respectively. Among all, ROBINA outperformed and has trade-offs compared with TANVEER, as shown in Figure 8 and Figure 9.

### 7.3. Analysis of Transmission Loss

**Figure 10:**
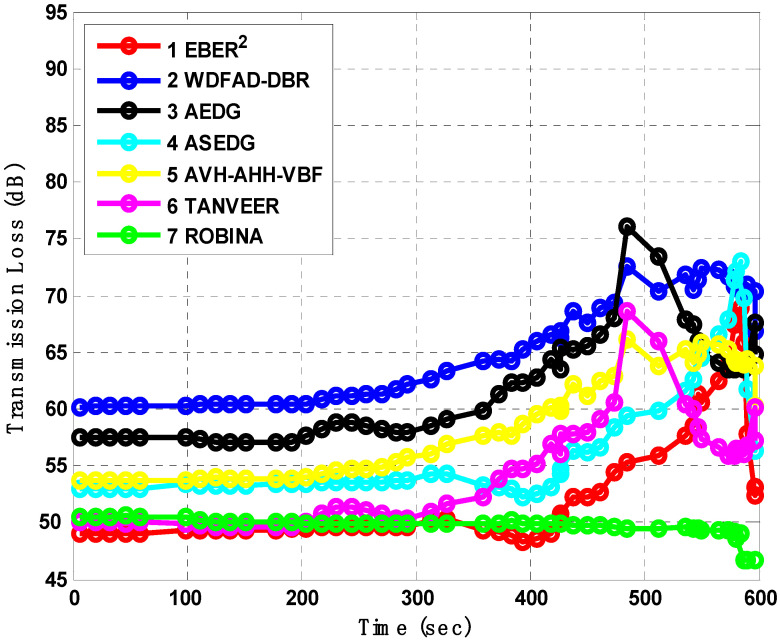
Analysis of average End-to-End delay with baseline schemes.

We also analyzed the mechanism of transmission loss (which is measured in dB) for intervals of 100 records iteratively, as shown in Figure 10. It is defined as the accumulated decrease in intensity of some network’s behavior to propagate through a certain area. This terminology is frequently used in acoustic scenarios. The following reasons are precuts for this, like the inadequate size of sensors, insulative of distributed sonobuoy low-power factors and weak acoustic links, (which are just mutual like systems that describe the acoustic performance because of large transmission loss underwater), including geometric spreading and sound absorption. Transmission Loss (TL) is a spatial-temporal variable in underwater acoustic channels; thus, it was measured for ROBINA to cross-check the performance of the proposed technique in large deep or shallow water. Ultimately, the TL was comparatively high in all similar schemes, at 50 dB for EBER^2^ and 50 dB for TANVEER. Both fired next forwarder nodes and Immediate Available Forwarder Nodes (I_AFN_) during packet transmission; TL was recorded for I_AFN_ (which is used by ROBINA) as approximately 49 dB, which outperformed all benchmark schemes. The minor differences observed in AEDG and ASEDG are due to the Ram nature of underline schemes. For AEDG, the sudden difference is out from 400 to 600 records and increased 55 to 75 dB due to delay and data from schemes. Although a great data gathering scheme, using AUV underwater, taking data from nodes and sending them to sink, is a lengthy process for AEDG and ASEDG. Additionally, WDFAD-DBR and AVH-AHH-VBF had values of 60 dB and 58 dB TL for 500 s until it reached 600.

### 7.4. Impact of Immediate Available Forward Nodes (I_AFN_)

**Figure 11:**
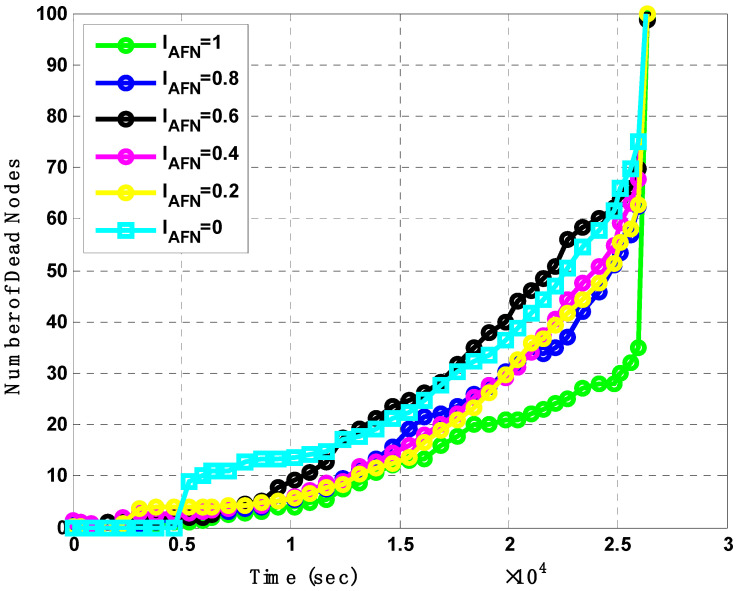
Analysis of Number of dead nodes with I_AFN_ and its effects.

In our proposed schemes, we also analyzed the effect of the involved parameter of I_AFN,_ which is based upon Algorithms 1 and 2 for ROBINA. The domain and range of this factor are taken from 0 to 1 with equal even intervals. To evaluate the performance of the I_AFN_, roughly 100 nodes were taken with 0 to 3 recode, though these are recorded for ten thousand. The proper scheme behaved equally when the number of I_AFN_ was between 0.6 to 0.8. For I_AFN_, at around 2.5 s the total was 38 number of dead nodes of ROBINA. Alternatively, the number of dead nodes increased with I_AFN_. Usually, the supportive factor in EBER^2^ is PFN, in AHH-AUV-VBR is VBF, and in WDFAD-DBR is DA division at the role of Member Nodes (MNs) in AEDG and ASEDG, respectively. Like TANVEER, the angled cone and Binary Inter Node (BIN-TANVEER) as supporting roles to map the relay nodes do not go to dead nodes. Therefore, it is closer to our proposed scheme. The following detachment of individual parameters with different periods is shown in Figure 11.

### 7.5. Analysis of Path Loss

**Figure 12:**
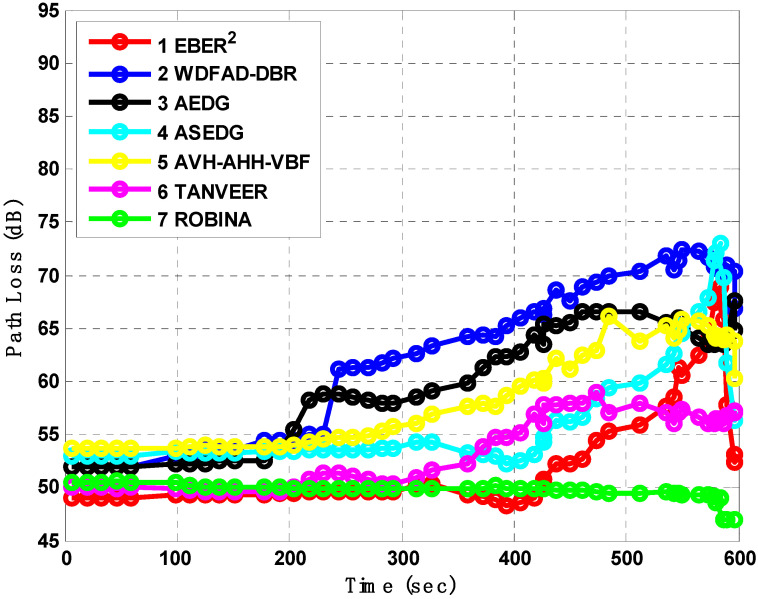
Analysis of path loss with baseline schemes.

The connection between the two nodes is established when the path remains active; especially in IoUTs, this is a vital concern when the node deploys in a dense environment. Usually, in these types of environments, nodes are particularly far apart from each other and not an intimation of path link breakage. These links suddenly create a void hole for a long time as holes become a void region that isolates the network. Due to the abovementioned reasons, the path analysis between all schemes was experimented and is shown in Figure 12. The WDFAD-DBR and AVH-AHH VBF tackled the depth and coped early to handle the harsh environment; therefore, their path loss was not much increased, only 50 dB and 52 dB, respectively. Rest schemes like EBER^2^, AEDG, and ASEDG lack such alternatives and, therefore, their path loss analysis satisfied 46 dB, 52 dB, and 53 dB, respectively. The TANVEER and ROBINA had path loss analysis around 200 s equally until it reached 400 s, at which point it increased to 50 dB and 51 dB, respectively. The 1 dB difference is ignorable after 5.5 min of simulation, by principle. Usually, this interval is considered good for out-breaking the result, especially when the critical parameter is under study. On the contrary, ROBINA started at 50 dB and reached the lowest path loss value even after 10 min equal to 600 s.

### 7.6. Analysis of DPD-TANVEER and PA-ROBINA with Number of Dead Nodes and Packet Transmitted

**Figure 13:**
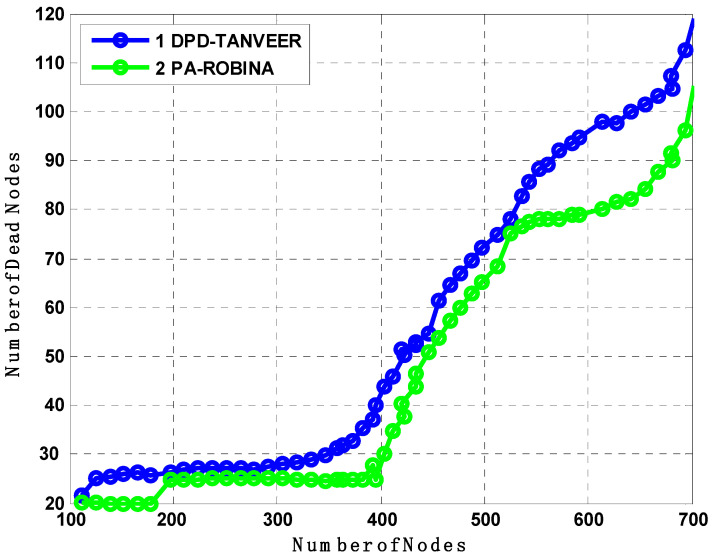
Analysis of the number of dead nodes compared with DPD-TANVEER.

It is evident from Figure 13 that the comparison between DPD-TANVEER (which is a sub-variant of TANVEER for delivery of data Packet, see [22]) and the sub-variant of our proposed approach PA-ROBINA (which is used for enhancing the node length to cover the maximum of relay nodes and I_AFN_) is represented. With time intervals, the performance of the network including the number of dead nodes was plotting. Initially, the benchmark approach of DPD-TANVEER had a smaller number of dead nodes until the first 50 s to 100 s, and the total number of dead nodes found less than one were ignorable. Suddenly, the next interval of 50 s, like in 150 s until it reaches 250 s, the number of nodes increased, which was eight nodes for PA-ROBINA and around ten nodes for DPD-TANVEER. During path adjustment, most of the nodes are easily covered under the radius of the destination.

**Figure 14:**
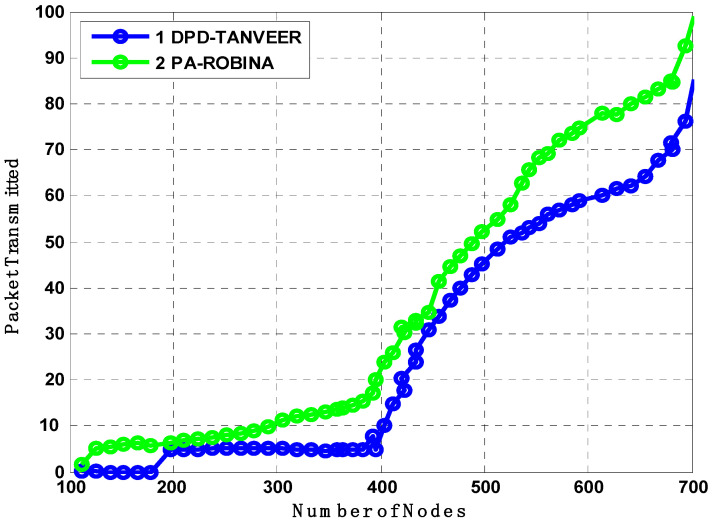
Analysis of the number of dead nodes compared with DPD-TANVEER and PA-ROBINA.

Furthermore, the PA-ROBINA also considered forwarder nodes distances; therefore, the closer nodes were easily covered. Meanwhile, in DPD-TANVEER, the speed purpose nodes are called watchman nodes, responsible for providing the immediate alternate rotation for dead nodes. As watchman nodes use some amount of energy, the time to recover and rescue the dead nodes is longer than ROBINA. As the number of dead nodes is decreased, the packed transmission is increased. It is earlier mentioned that the procedure of both TANVEER and ROBINA techniques, therefore, consider and analyze the effect of Figure 13 on Figure 14. Although, the packet transmission of PA-ROBINA is majorly increased and dominant over DPD-TANVEER.

### 7.7. Analysis of DPD-TANVEER and PA-ROBINA with Energy Tax

**Figure 15:**
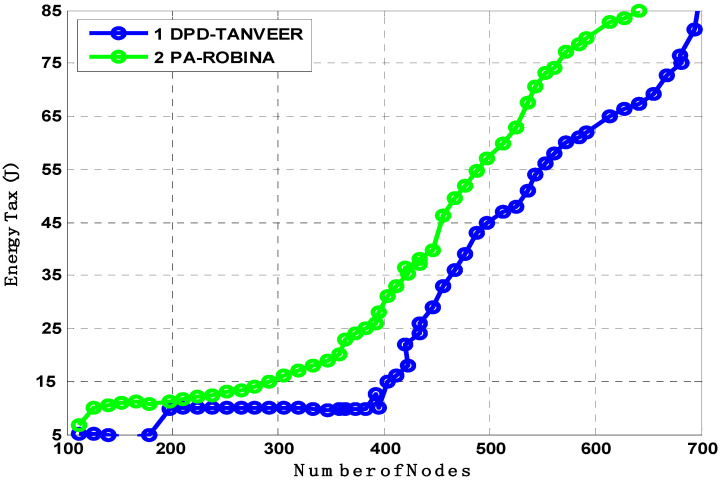
Analysis of energy tax compared with DPD-TANVEER and PA-ROBINA.

The amount of EC was experimented with and shown in Figure 15 between ROBINA with its other selected similar state-of-the-art schemes. Energy is the fundamental parameter in all routing protocols, even where the unmanned efforts requiring the replacements of nodes or batteries are much more difficult. Due to the atomic path with its MNs used in AEDG and ASEDG, EC is higher than WDFAD-DBR and EBER^2^, which we did not consider in experiments because ROBINA used the capacity of internodes adjustment and had a mechanism to avoid void holes. Hence, it saved the most energy among all routing schemes when it was used.

Energy is the primary restriction in all routing strategies regardless of the strategy or technique employed. As a result, Figure 15 compares the energy usage of DPD-TANVEER and PA-ROBINA. During the transfer of packets from source to destination, energy tax analyses were performed. When the network was not dense, the tax was not eaten as much as in DPD-TANVEER; because TANVEER skips the empty areas and sends the data packet directly to the runabout, it is slightly slower than PA-ROBINA. With DPD-TANVEER, the least potential energy consumption was counted at 0.6 J for 150 nodes and 0.8 J when nodes approached their limit. This is due to triangle-baud measurement when the angled cone is determined for the data packet’s destination through all directions. It is interesting to note that the DPD-TANVEER was comparatively high when simulated with L2-ABF, though still somewhat lower (i.e., slightly lower) when compared to PA-ROBINA. The adjustment and consumption method for relay nodes and (I_AFN_) was slightly better than its data delivery; because of the large-scale network (we employed 800 nodes in ROBINA and 200 nodes in TANVEER), the IoUTs are frequently unpredictable, especially regarding energy tax. The cost of node mobility in this scenario is not a positive factor, and the amount of energy is purposely loaded. Meanwhile, the ROBINA outperformed in these scenarios and covered both (water/node movement, frequently charged topology, dynamic and static sinks stations, acute communication, and water current, which is also unpredictable with dense to shallow water) using the rotation-based path and mechanism of inter-node adjustment. When a medium to the large-scale network was eliminated as a text-bed, the amount indicated by our technique was not debatable; [34,35,36,37,38,39] is a good example for this understanding.

### 7.8. Analysis of BIN-TANVEER and PL-ROBINA with Number of Dead Nodes and Packet Transmitted

**Figure 16:**
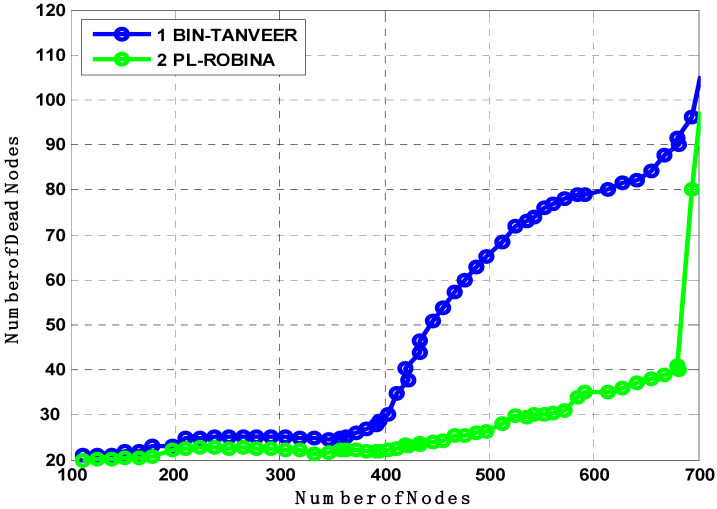
Analysis of the number of dead nodes compared with BIN-TANVEER and PL-ROBINA.

**Figure 17:**
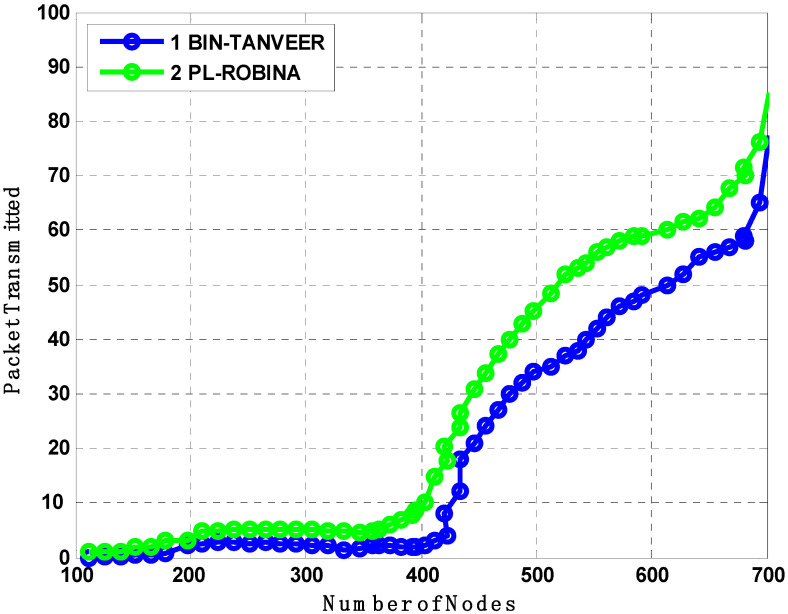
Analysis of packet transmission compared with BIN-TANVEER and PL-ROBINA.

**Figure 18:**
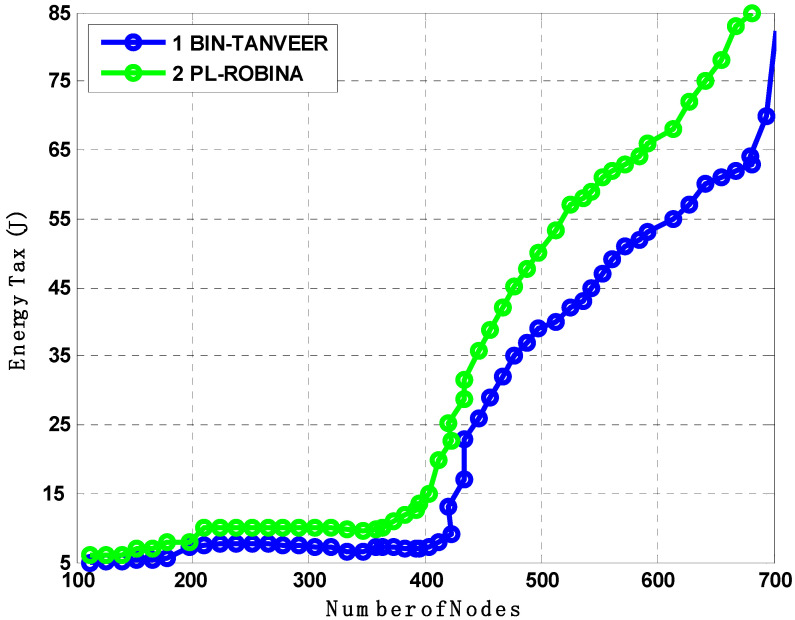
Analysis of energy tax compared with BIN-TANVEER and PL-ROBINA.

The BIN-TANVEER and PL-ROBINA relation is presented the same scenario of finding the number of dead nodes in Figure 16. The path-loss mechanism is supported by *Urick*’*s model* and the *Colored Gaussian*’*s Surface (CGS)* for optimizing the routing path and acute signal between two communicated nodes, so the path loss is dubbed according to Equation (1). Typically, with the use of the practical value of ‘*K*’ with ‘*Throp*’*s formula*’ for derivative by integration, it has been observed that the existing monitoring IoT system with some routing mechanism ignores the factor of path loss (here, we considered the path loss between only two communicated IoT devices (whatever the way underwater) and the main concern was its recovery as well as detection to find out the health of the network when the path was not found, for the time being, between two devices) to ensure if it recovers from two connected devices and if recovery is so for all devices (connected) in the routing path by the help of ‘*Tylor and Maclaren Series*’ that already used in sensor data cryptography [40,41,42]. Therefore, the path loss is best described in the PL-ROBINA scheme over BIN-TANVEER. Although the PL-ROBINA outperformed even the largest number of nodes (say 750 from Figure 16), the BIN-TANVEER has only handled the nodes and their dead quantity with suitable energy tax when the network was in the category of the small to medium scale, as contrary to PL-ROBINA for medium to large-scale networks. Ultimately, the packet transmission is also attached as for the energy tax from Figure 17 and Figure 18. From Figure 17, the performance of PL-ROBINA is satisfactory as evidenced by its large number of packets transmitted due to the non-loss of the path between two connected devices; BIN-TANVEER cannot handle a large amount of data, as the Binary Inter Nodes (BIN’s) are not functional enough and supported with this kind of situation. It is only built on purpose in TANVEER, and as mentioned in [22], the packet success ratio achieved 94% of ROBINA and AVH-AHH-VBF. In addition, the WDFAD-DBR has the least ratio, which is only 62%. Meanwhile, the same situation will be observed for energy tax with BIN-TANVEER and PL-ROBINA respectively.

## 8. Conclusions

The void hole problem has a long-lasting effect in the acoustic environment that attracts lots of attention from the research community. In order to address this problem for the sake of performance analysis and evaluation, we performed extensive simulation against the different number of dead nodes: packet transmission, energy tax, transmission loss, and path loss mechanism with state-of-the-art techniques like EBER^2^, AEDG, ASEDG, WDFAD-DBR, AVH-AHH-VBF, and TANVEER. Simulated results showed that our proposed scheme performed better to increase high packet transmission and minimum transmission loss and path loss with affordable E2E delays. Therefore, we offered a novel avenue that has rotational orbits based on its mechanism of inter-node adjustment that has affordable E2E, which is 0.2 and remains constant. The EC and network lifetime also had good simulation results due to their path adjustment (that had orbital shapes) mechanisms. We also improved the packet transmission through this scheme around 68–92%, even in a dense network. Comparatively, all other schemes were not yet experimental when the network was dense (say 500 number of the node). Overall, we can conclude that the ROBINA outperformed against EBER^2^ and WDFAD-DBR in terms of E2E and transmission loss, which was 60% higher than the abovementioned. Moreover, the AEDG and ASEDG were experimental against packet success ratio and path loss the proposed scheme, ROBINA, have similar results due to the dense avoidance of void hole problems compared with TANVEER and its variants. In the future, we have the intention to simulate ROBINA and propose a similar technique that enables blockchain technology for very large-scale networks with a testbed, large oceanic environment.

## Figures and Tables

**Table 1:** Acronyms and their descriptions.

*Acronym*	*Description*
***Selop***	***Selection operator***
***B***	***Beacon message***
***Dn***	***Number of dead nodes***
***SNN***	***Sink neighboring nodes***
***Ns***	***Set of neighboring nodes***
***Sn***	***Set of nodes***
***PACK***	***Packer acknowledgment***
***AdjNi***	***Adjacency of node***
***Node location* (*N_l_*)**	***Node location***
***E*(*Ni*)**	***Energy of node***
***SNN*(*_range_*)**	***Range of sink neighboring nodes***

**Table 2:** Simulation settings and their value.

Simulation Parameters	Values
Number of nodes	700
Network Range	1600 × 1000 × 1000
Initial energy	100 j
Acoustic Network Speed	1500 m/s
Transmission range	200 m
Receiving	0.8 W
Data packet size	50 B
Beacon message size	52 B
Data Rates	10 kbps
Network Simulator with AquaSim	NS-2.35

**Table 3:** Overall performance trade-offs.

Schemes	Features	Achieved Parameters	Trade-Offs
***ROBINA***	First time introduce the path rotation idea with path loss and adjustment variants in the acoustic environment.	Avoidance of path loss and increased the reliability of energy-efficient routing that supports IoT-based dynamic devices.	The maximum probability of path rotation and internodes adjustment is achieved with affordable AE2ED
***PA-ROBINA***	Adjustment is easy, avoidance of void hole is minimal, and rotatable in a dynamic environment	Path adjustment is performed against different distances and cross-checks for I_AFN._ PA-ROBINA has more satisfactory results then DPD-TANVEER	Observed good results when compared with similar approach DPD-TANVEER and average results with rest of the schemes
***PL-ROBINA***	Using Urick’s Model ad Colored Gaussian Surface with Throp’s Formula, the PL-ROBINA is established between two nodes and remains true for all other nodes with reference to ‘*Tylor and Maclaren Series*’.	From Figure 12, path loss analysis had good results and easily experiment with the medium to large scale networks even 700 nodes	This scheme is only supported when taking the assumptions of mathematical models; otherwise, it is needed to try with small networks
***TANVEER*** [22]	Geographic and opportunistic routing scheme using three-angle adjustment and watchman-based transmission	Increased PDR and throughput of the network by bypass the empty nodes/regions	Observed high end-to-end delay due to three-time angle calculation
***LBA***- ***TANVEER*** [22]	Layer-based adjustment with data collision avoidance mechanism	Improved network topology and performance with adjustment of nodes	Due to the dynamic nature of the environment but accurate void nodes are feasible
***DPD***- ***TANVEER*** [22]	Using the TANVEER approach with avoiding empty regions	Improved PDR, throughput, and a fraction of empty regions	Same as TANVEER
***BIN***- ***TANVEER*** [22]	Works with Binary internodes that rescue the data transmission	Improved PDR and try to decrease PLR	Energy consumption is high
***EBER*^2^** [14]	deliberates enduring energy along with the number of PFN transmission choices duplication of packets, residual energy	balance energy and achieve reliability	The protocol allows forwarders to adaptively control their communication according to the utmost node in the neighbor list of the network only
***AEDG*** [13]	AEDG and ASEDG both introduce the atomic shape path for relay nodes and sinks, but it is not rotational	need to design such a routing path that is not only shortest in length but also covers realistic parameters like energy tax, AE2ED	The atomic path is not as much rotated to cover the case; the average results are shown in Figure 10, Figure 11, Figure 12 and Figure 13, respectively
***ASEDG*** [11]	AEDG and ASEDG both introduce the atomic shape path for relay nodes and sinks, but it is not rotational	routing path not only shortest in length but also cover the dead nodes, further using AUVs is not a smart approach, especially when the network is large	The atomic path is not as much rotate to cover the case [40]; the average results have shown in Figure 10, Figure 11, Figure 12 and Figure 13, respectively
***WDFAD****-**DBR*** [15]	mechanism considers the depth of the next forwarding node through which it avoids void holes.	With DBR joined with this. Therefore this scheme is a benchmark, so it works well for rapid data delivery as compared with other schemes	Depth is not the solution in any case; the one using depth Division is considered a traditional approach that only fits when nodes are not far apart from each other
***AVH****-**AHH**-**VBF*** [9]	schemes have been designed including hops mechanism, Vector-Based Forwarding	AHH-VBF protocol, each node uses dissimilar virtual pipes, and during each time of transmission direction of the virtual pipe change	SM-AHH-VBF, without this feature, the result has been affected
***PSOA*** [43]	Decision feedback equalizer (DEF), ambient noise, Laplace noise, Raley Fading channel, and distribution for frequency selection in the acoustic environment is used	The computational complexity of LMS, RLS, and PSO are achieved	PSO has the highest computational complexity as compared with PSO-DEF
***Underwater OFDM Reciver*** [44]	Design the simplifier receiver for the UWA channel due to deep neural network-based orthogonal frequent division multiplexing	Thus the signal is suitable for UWA and also for other similar schemes and channels due to better bit error over traditional ones	A general receiver that is already used for other modulation schemes, not fit for water currents, particularly when the water is deep and shallow
***EACQ for Underwater*** [45]	Environment-aware communication channel quality prediction (ML-ECQP) method for UACNs is proposed	A logistic regression algorithm is used to predict the communication channel quality between the sender and receiver side	Highly energy waste caused transmission that reduces the packet transmission ratio
***OCRPC for IoUTs*** [46]	Try to evaluate the optimal transfer power node for deleting of nodes to maximize the PDR and other relevant parameters, for example, Q-Network based underwater relay section as well as Q-learning approaches	Achieved equal transmit power under the same condition using Markov Model and reinforcement learning	Improve the communication strategy, but lots of iterations are needed to cover the maximum area
***RCACR*** [47]	Same as used reinforcement learning mechanism as per [46], the only difference is that accelerating the convergence algorithm strategy is introduced for the first time in literature in underwater settings	Due to the reward function with reinforcement learning mechanism, results are easily satisfied in the MAC layer	Due to the proposed modified MAC layer in Underwater to optimal routing decision, the optimal RCACR pour performed HHVBF, DQELR, and GEADR in terms of convergence and energy
***DQELR*** [48]	adaptive Deep Q-Network-based energy- and latency-aware routing protocol (DQELR) to increase the network lifetimes in Underwater	Achieved less limitation in latency and increased energy efficiency with superior network lifetime	The method of selecting Q-value is not optimized; it is proactive and does not fit in all scenarios that the case is covered
***ML Algorithms for Underwater*** [49]	Adaptive modulation and coding mechanism is used for 3D trail data set for oceans	Boosted regression tree and four ML algorithms are good for channel characteristics	SNR and BER constraints are rich; especially signal characteristics are used
***Fuzzy decision Making in Underwater*** [50]	Packet forwarding scheme using fuzzy logic is introduced, with RSSI indicator for adaptive and non-adaptive transmission	Several hops with fuzzy constraints are used that dynamically affect the performance of the underwater networks, especially energy tax	Hop count is an old parameter, but coins with fuzzy logics decision making structures are good to introduce acoustic communication
***Miscellaneous Underwater Routing Schemes*** [51,52,53,54,55,56]	All are working for underwater scenarios to avoid and solve the void hole problems	Proposed atomic path, watchman-based nodes, optimal scheme, angle adjustment, diagonal and vertical routing ideas, and different comparative studies work well in this domain	All the routing schemes were well performed in and deployed in a network scenario with some trade-off’s relationships

## Data Availability

Not applicable.
